# Identification of a major causative agent of human cercarial dermatitis, *Trichobilharzia franki* (Müller and Kimmig 1994), in southern England and its evolutionary relationships with other European populations

**DOI:** 10.1186/1756-3305-7-277

**Published:** 2014-06-19

**Authors:** Scott P Lawton, Rivka M Lim, Juliet P Dukes, Richard T Cook, Anthony J Walker, Ruth S Kirk

**Affiliations:** 1 Molecular Parasitology Laboratory, School of Life Sciences, Kingston University, Kingston upon Thames, Surrey KT1 2EE, UK

**Keywords:** *Trichobilharzia franki*, Cercarial dermatitis, UK, Re-emerging disease, 28S ribosomal DNA, ITS1 haplotypes

## Abstract

**Background:**

*Trichobilharzia* is the most species rich and widely distributed genus of schistosomes and is known throughout Europe and North America as an agent of human cercarial dermatitis. The disease is caused by an acute allergic reaction in the skin that develops as a consequence of repeated contact with water containing schistosomatid cercariae. However, despite historical outbreaks of the disease, there are no published records of accurately identified *Trichobilharzia* species from the UK.

**Methods:**

Two hundred *Radix auricularia* (L.) were sampled from a recreational fishing lake in Hampshire and emerging schistosomatid cercariae were collected for microscopy and DNA extraction. General morphological description of the cercariae was performed, alongside sequencing and phylogenetic analysis of the 28S ribosomal DNA for accurate species identification as well as comparisons of ITS1 in order to identify evolutionary affinities with other European populations. All molecular comparisons were performed using published sequences.

**Results:**

The phylogenetic analysis of 28S sequences identified the cercariae as *Trichobilharzia franki*. Two unique British ITS1 haplotypes were identified which were most closely related to haplotypes of *T. franki* populations from France. Haplotype network analysis indicated the mixing of *T. franki* populations throughout Europe. It is suggested that parasite distribution is the probable result of the movement of migratory waterfowl.

**Conclusions:**

This is the first accurate record of *T. franki* in the UK. The movement of *T. franki* with waterfowl could pose a considerable human health risk, as in mainland Europe, and signifies *T. franki*-associated human cercarial dermatitis as a re-emerging disease in the UK.

## Background

The cercariae of many species of avian schistosomatid blood flukes are known to cause human cercarial dermatitis (CD), also known as swimmer’s itch. Avian schistosomatids have a truly global distribution with agents of CD being recorded on all major continents except Antarctica. However, throughout Europe and America there has been increased incidence of reported infections and CD is now considered an emerging and re-emerging infectious disease [1-3]. Foci of infection have been identified in several European countries particularly Italy, Germany, Austria, Switzerland, the Netherlands, Czech Republic, France, Poland and Iceland with the disease impacting on local economies that depend on tourism associated with recreational water use [4].

Despite the occurrence of these parasites throughout Europe and North America [1,3], relatively little is known about the occurrence of CD and the identity and diversity of parasites that cause it in the UK, even though outbreaks of CD have been recorded in Loch Lochore in Fife in 2006, the Norfolk Broads in 2004 [5], a water sports lake in Suffolk in 1987 [6], a leisure water park in Rickmansworth, Hertfordshire in 1970 [7] and Roath Park, Cardiff between 1928–1943 [8].

Human CD presents as an acute allergic reaction in the skin that develops as a consequence of the repeated penetration of certain species of schistosomatid cercariae that emerge from freshwater snail intermediate hosts [3,9]. Maculo-papulo-vesicular eruptions occur after exposure, followed by intense itching, fever, swelling of the lymph nodes and eventually erythema and oedema [10]. Experimental studies on immunocompetent mammalian models show that although the majority of the cercariae die in the skin, some schistosomula can migrate to the lungs (visceral avian schistosome species) or nervous system (*Trichobilharzia regenti*) [3,9]. Severe pathologies can develop in immunodeficient mammals due to a high number of schistosomula migrating to organs. Therefore, although they are unable to complete their development and reproduce, the parasites may cause pathologies in addition to those associated with the skin (reviewed by Kolářová [9]).

*Trichobilharzia*[11] is considered the most species-rich and widely distributed genus of the Schistosomatidae, with species parasitizing waterfowl throughout the world, and several being leading causes of CD [1]. Schistosomatids in UK waters have been inadequately studied. Although ocellate schistosomatid cercariae have been recorded from snails in the UK [8,12,13], there is no definitive identification of a *Trichobilharzia* species. In the last decade, problems relating to the accurate morphological identification of *Trichobilharzia* species associated with CD outbreak sites in America and Europe have been overcome by using ribosomal DNA (rDNA) markers to identify cercariae to species [1,14]. Such approaches were employed in the current study to identify schistosomes released by aquatic lymnaeid snails collected from Tundry Pond, Hampshire. We report the first detailed record/description of *T. franki* Müller and Kimmig [15] in the UK and identify evolutionary relationships with other European populations of this schistosome. The public health implications of the findings are discussed in the context of CD being a re-emerging infectious disease in the UK.

## Methods

### Snail and cercariae collection

Two hundred *Radix auricularia* (L.) were collected and identified based on shell morphology from Tundry Pond, a recreational fishing lake in Hampshire, Southern England, UK (grid reference: SU 775 525, 51.16 N 0.53 W), in August 2011 as part of an on-going study on the diversity of digeneans and their intermediate hosts in the UK. Ocellate furcocercariae have previously been observed at this site (R. Kirk, unpublished observations). Snails were relocated to the laboratory for processing. Each snail was isolated in a 100 ml beaker filled with dechlorinated tap water that had been filtered through a Brimak carbon filter (Silverline UK) and cercarial emergence was stimulated by exposure to natural light. Three *R. auricularia* shed ocellate furcocercariae. These cercariae were collected for molecular work using the techniques described by Brant and Loker [1] and were pooled in Petri dishes for morphological description. Upon confirmation of species, parasite and snail reference material were deposited at the Natural History Museum, London, UK (parasite voucher NHMUK 2014.4.25.1-2; snail voucher NHMUK 20140076).

### Morphological description of cercariae

Live cercariae were vitally stained with 0.5% neutral red and examined using light microscopy for initial morphological description. To obtain further morphological information, cercariae were fixed for scanning electron microscopy in 2.5% glutaraldehyde in 0.1 M phosphate buffer (pH 7.4) for 2 h at 4°C. They were then washed in four changes of 0.1 M phosphate buffer, post-fixed in osmium tetroxide in the same buffer for 1 h, dehydrated in a graded ethanol series and dried in hexamethyldisilazane. Samples were coated with gold-palladium and examined under a Zeiss EVO50 scanning electron microscope. Morphometric parameters of cercariae were not recorded because of the unreliability of using cercarial dimensions and flame cell organization for identification of *Trichobilharzia* species [10]. Chaetotaxy was not performed due to known problems with misinterpretation of sensory structures [16] and staining [16,17].

### Molecular identification of parasites

Live ocellate furcocercariae were morphologically identified to genus level using light microscopy and were pooled into 1.5 ml microfuge tubes (50 cercariae per snail). Each tube was centrifuged at 10000 rpm for 1 min to pellet the cercariae and DNA was extracted with the Qiagen DNeasy blood and tissue kit (Qiagen Inc.) using the manufacturer’s protocol. PCR was performed to amplify fragments of 28S ribosomal subunit (LSU) and the internal transcribed spacer region (ITS) using primers and cycling conditions specified by Olson *et al*. [18] and Wang *et al*. [19], respectively. PCR reactions were performed using 12.5 μl Thermo–Start^R^ PCR master mix (0.625 Units of Taq DNA polymerase, 1X reaction buffer, 0.2 mM of each dNTP and 1.5 mM MgCl_2_) and 1–2 ng/μl of DNA. Final reactions were made up to 25 μl with PCR-grade water. Reactions were performed using a Veriti 96 well thermal cycler (Applied Biosystems™) and 5 μl of each amplicon was visualized in 1% agarose gels stained with gel red (Bioline™). The remaining 20 μl PCR products were sequenced at the DNA sequencing facility of the Natural History Museum, London, using the PCR primers with Fluorescent Dye Terminator Sequencing Kits (Applied Biosystems™); sequencing reactions were run on an Applied Biosystems™ 3730XL automated sequencer. Resultant sequences were assembled using BioEdit [20] and manually corrected for ambiguous base calls. It is important to note that despite the DNA being extracted from pooled cercariae, there were no polymorphic sites noticed in the chromatographs of both the forward and reverse sequences of LSU and ITS. Complete sequences were submitted for BLAST search using blastn (http://blast.ncbi.nlm.nih.gov/) to enable initial identification, indicating that all the sequences generated best-matched *T. franki*. Novel LSU sequences were aligned with previously published sequences of other species of *Trichobilharzia* and bird schistosomes from different genera and *Schistosoma sinensium* as an out group (Table 1) using MUSCLE (http://www.ebi.ac.uk), giving an alignment of comparable data of 556 bp.

**Table 1:** **Published sequences of schistosomatids used in the phylogenetic analysis of the LSU to identify UK specific *Trichobilharzia *** **species**

**Species**	**Host**	**Accession**
*Trichobilharzia franki*	*Radix auricularia*	HM131141
*T. regenti*	*Anser anser*	HM439491
*T. querquedulae*	*Anas discors*	FJ174469
*T. physellae*	*Physa parkeri*	FJ174475
*T. szidati*	*Lymnaea stagnalis*	FJ174476
*T. stagnicolae*	*Stagnicola emarginata*	FJ174479
*T. ocellata*	*Lymnaea stagnalis*	AY157243
*Anserobilharzia brantae*	*Chen caerulescens*	FJ174467
*Dendritobilharzia pulverulenta*	*Gallus gallus* (Lab host)	AY157241
*Gigantobilharzia huronensis*	*Agelaius phoeniceus*	AY157242
*Heterobilharzia americana*	*Mesocricetus auratus*	AY157246
*Schistosomatium douthitti*	*Mesocricetus auratus*	AY157247
*Ornithobilharzia canaliculata*	*Larus delawarensis*	AY157248
*Austrobilharzia terrigalensis*	*Batillaria australis*	AY157249
*Bilharziella polonica*	*Anas platyrhynchus*	AF184265
Out group		
*Schistosoma sinensium*	*Mus musculus* (Lab host)	AY157251

### Inter- and intra-species phylogenetic analysis

To accurately identify the novel sequences and place them into a phylogenetic context with other species of *Trichobilharzia*, phylogenetic analysis was performed for alignments of LSU using MEGA5 [21]. Several phylogenetic methods were employed using the neighbour joining (NJ) approach under the conditions of the Jukes-Cantor model, maximum likelihood (ML) methods using the HKY+G model of evolution also identified in MEGA5 [21]. Maximum parsimony (MP) trees were obtained using the close–neighbour–interchange algorithm. The model used for the ML tree was identified based on the lowest Bayesian information criterion scores relative to the other models tested and for all tree analysis nodal support was assessed using 1000 bootstrap replicates. The pair-wise uncorrected distances (*p-*distance) were also calculated for the LSU sequences between *Trichobilharzia* species to estimate genetic distance and to validate species identification.

In order to establish affinities between European and UK isolates of *T. franki*, ITS1 fragments were analysed due to the availability of comparable data from other European *T. franki* populations. The three novel sequences obtained were aligned with previously published sequences that represent populations of *T. franki* from across central and northern Europe, producing an alignment of 878 bp. Sequences were aligned and phylogenetic analysis performed as described previously, but ML trees were constructed using the K2+G model as identified by MEGA5 [21]; *T. regenti* was used as an out-group in all analyses. Standard population genetic analysis was performed using DnaSPv5 [22] to calculate nucleotide diversity (π) and sequence differentiation within and between sequence sets from different countries and to identify the occurrence of unique haplotypes (H) and haplotype diversity (Hd). In order to assess the association of the UK haplotypes to others from Europe, the most parsimonious haplotype network was constructed using TCS version 1.13 [23].

## Results

### Morphological description of *Trichobilharzia franki*

**Figure 1:**
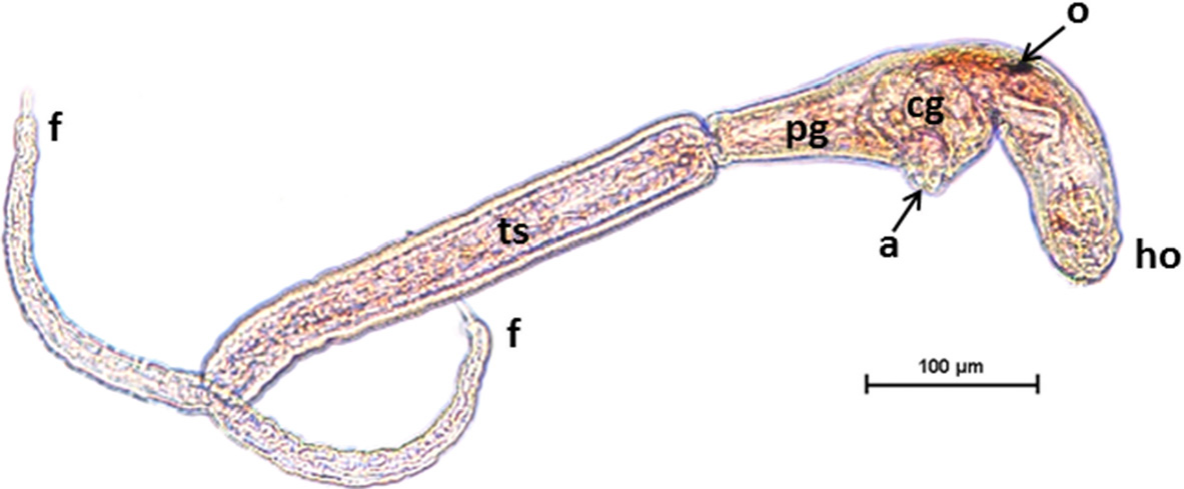
**Lateral view of the cercaria of *Trichobilharzia franki *stained with neutral red.** Acetabulum (a), head organ (ho), one of two ocelli (o) circumacetabular penetration glands (cg), postacetabular penetration glands (pg), tail stem (ts) and furcae (f).

**Figure 2:**
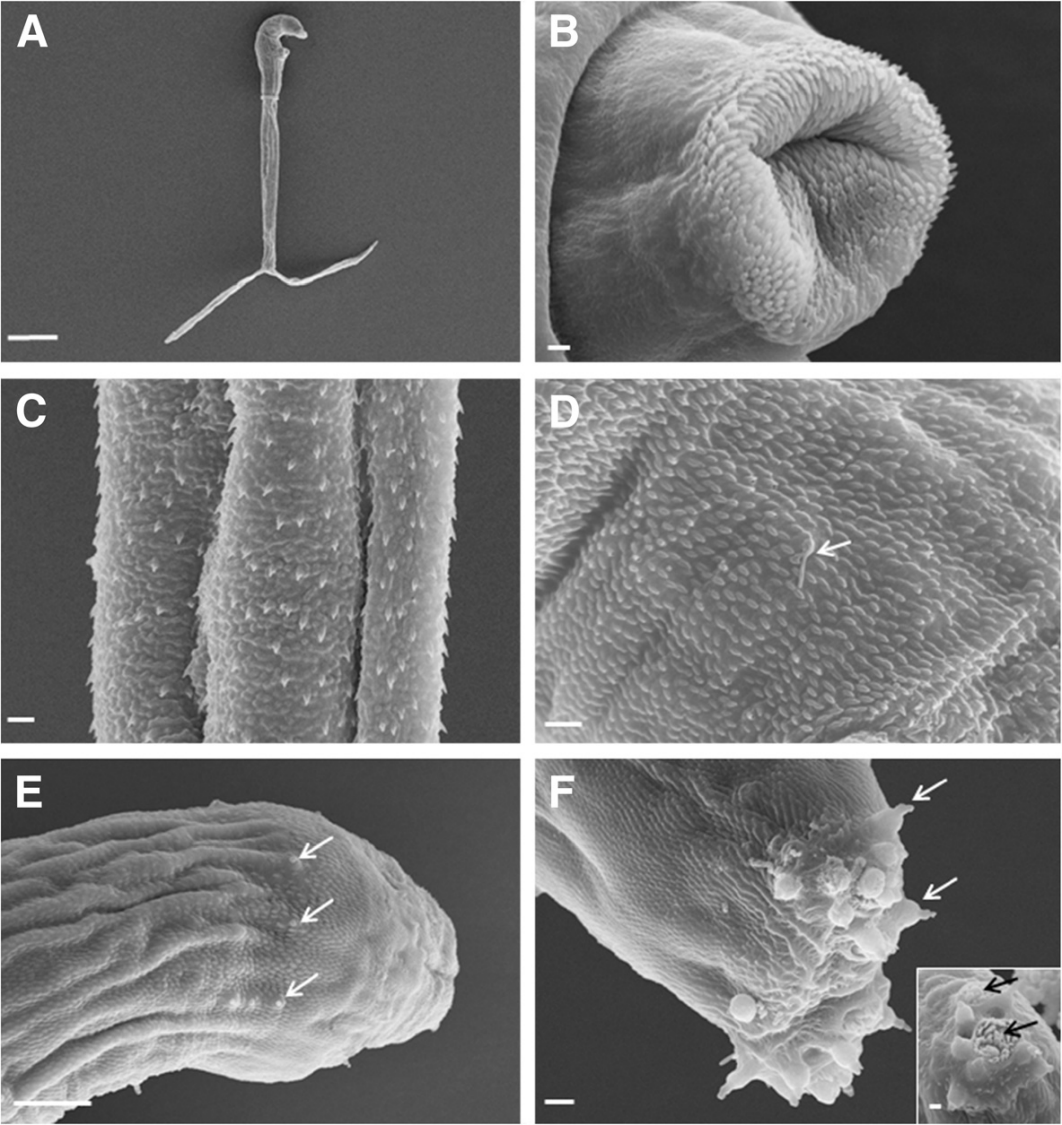
**Scanning electron micrographs of the cercaria of *Trichobilharzia franki*. A**. Entire cercaria, scale bar = 100 μm. **B**. Acetabulum, scale bar = 1 μm. **C**. Tail stem with spines, scale bar = 2 μm. **D**. Tegumental spines and type 2 sensory papilla on body (arrow), scale bar = 2 μm. **E**. Anterior of body and type 1 sensory papillae (arrows), scale bar = 10 μm. **F**. Apical view of body showing head organ, note type 1 sensory papillae (arrows) scale bar = 2 μm, and gland duct openings (insert, black arrows), scale bar = 1 μm.

The morphology of the ocellate furcocercariae partly corresponded with the previous description of *T. franki* by Müller and Kimmig [15]. The ocelli (eye spots) were located on the dorsal part of the body, between the acetabulum and the head organ. The head organ and the acetabulum were spherical. The cercariae were apharyngeate, with a subterminal mouth and a bifurcate intestine, which were inconspicuous. The body contained five large pairs of penetration glands, two pairs circumacetabular and three pairs postacetabular, in addition to an apical gland group, with all ducts opening anteriorly. The excretory system progressed through the excretory junction at the base of the body and extended into the tail stem to the tip of the furcae. The furcae were surrounded by finfolds and ended in thorns (Figures 1 and 2A). The description of the head organ by Müller and Kimmig [15] as a fused complex of oral sucker and pharynx and the preacetabular position of two pairs of penetration glands are suggested to be errors. These authors also reported that the tegument was covered with small spines. Here, scanning electron microscopy revealed that most of the external surface of the acetabulum was devoid of spines and only the inner surface had centrally directed curved spines with rounded tips (Figure 2B). The spines on the tail stem were large and curved (Figure 2C). The spines on the body resembled those on the acetabulum (Figure 2D). All spines were posteriorly directed. Two types of uniciliate sensory endings were observed: type 1 sensory papillae (with tegumental collar) of different lengths on the body and type 2 sensory papillae (without tegumental collar) of different lengths on the tail stem, furcae and body, but not on the head organ region (Figure 2D, E), corresponding to the description for *T. franki* by Kock and Böckeler [16]. The tip of the head organ displayed gland duct openings surrounded by type 1 sensory papillae (Figure 2F).

### Molecular identification of *Trichobilharzia franki*

**Figure 3:**
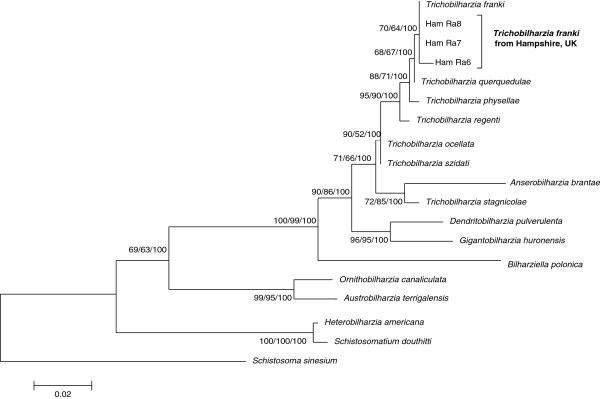
**Phylogenetic tree based on partial LSU sequences for avian schistosomes in order to identify ocellate furcocercariae from Hampshire, UK.** The samples sequenced from Hampshire, UK have close affinity with *T. franki* and do not associate with another species. The tree was constructed using the ML method in MEGA5 using the HKY + G substitution model. The scale shows the number of nucleotide substitutions per site between DNA sequences and nodal supports were generated using 1000 bootstrap replicates. The nodal support is given in NJ, ML and MP bootstraps respectively and only values >50 for at least two of the analyses are shown.

Phylogenetic analysis of the *T. franki* LSU fragments with 15 other schistosomatids produced a well-supported *Trichobilharzia* clade with *Dendritobilharzia pulverulenta* and *Gigantobilharzia huronensis* forming a sister clade (Figure 3). Within the *Trichobilharzia* clade the sequences generated in this current study, Ham Ra6 – 8 (GenBank: KJ775865-67), clustered with *T. franki* only and formed a shallow, but well supported, clade separate from any of the other taxa (Figure 3). This relationship between the UK sequences and *T. franki* was further supported by the low *p*–distance values relative to comparisons with other species (Table 2). However, a noteworthy observation is that similar levels of genetic divergence seen between the UK specific sequences and *T. franki*, were also evident between *T. franki* and *T. querquedulae* (Table 2). *Trichobilharzia querquedulae* appears as a sister species to *T. franki*, but short branch lengths may indicate that both species are either subspecies or members of a species complex.

**Table 2:** **Uncorrected pair-wise distance (p–distance) between different species of *Trichobilharzia *** **based on number of base differences per site between sequences of LSU**

**Species**	***T. stagnicolae***	***A. brantae***	***T. szidati***	***T. ocellata***	***T. regenti***	***T. physellae***	***T. querquedulae***	***T. franki***	**Ham Ra6**	**Ham Ra7**
***T. stagnicolae***										
***A. brantae***	0.037									
***T. szidati***	0.015	0.040								
***T. ocellata***	0.015	0.040	0.000							
***T. regenti***	0.024	0.050	0.009	0.009						
***T. physellae***	0.026	0.051	0.011	0.011	0.009					
***T. querquedulae***	0.024	0.050	0.009	0.009	0.007	0.006				
***T. franki***	0.026	0.051	0.011	0.011	0.009	0.007	0.002			
**Ham Ra6**	0.029	0.055	0.015	0.015	0.013	0.011	0.006	0.004*		
**Ham Ra7**	0.026	0.051	0.011	0.011	0.009	0.007	0.002	0.000*	0.004*	
**Ham Ra8**	0.026	0.051	0.011	0.011	0.009	0.007	0.002	0.000*	0.004*	0.000*

Those numbers highlighted by* are indicative of the *Trichobilharzia* species sampled from the UK (Ham Ra6 – 8) and their confirmation as *T. franki* when compared to other species.

### Insights into the relationships between populations of *Trichobilharzia franki* from the UK and Europe

**Figure 4:**
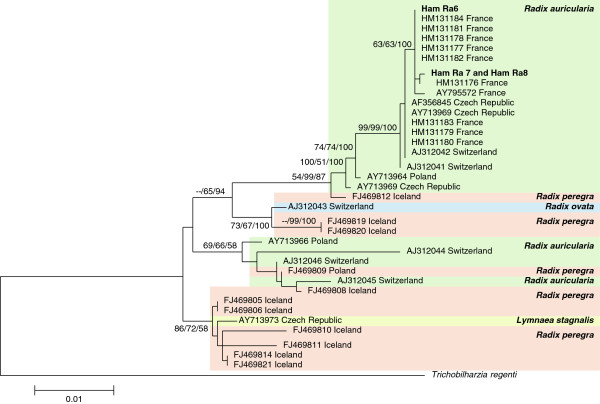
**Phylogenetic tree based on the ITS1 sequences representing different populations of *Trichobilharzia franki *from European countries and the snail species that they infect.** The samples sequenced from Hampshire, UK cluster within a France-specific clade, the only country specific clade to emerge during analysis. The tree was constructed using the ML method in MEGA5 using the K2 + G substitution model. The scale shows the number of nucleotide substitutions per site between DNA sequences and nodal supports were generated using 1000 bootstrap replicates. The nodal support is given in NJ, ML and MP bootstraps respectively and only values >50 for at least two of the analyses are shown.

**Figure 5:**
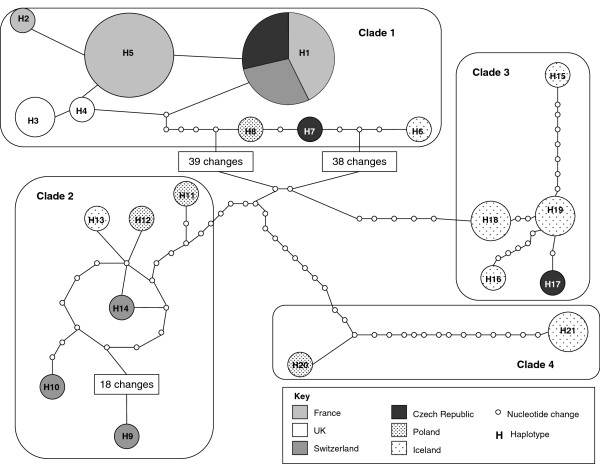
**Network of ITS1 haplotypes of *Trichobilharzia franki *from Europe.** Each circle represents a single unique haplotype and the size is proportional to the number of sequences with that specific haplotype. The ITS1 shows the UK sequences to have unique haplotypes (H3 and H4), but to be most closely related to haplotypes from France (H2 and H5) with only 1 to 2 nucleotide differences separating them. There is also some evidence of gene flow between geographical locations with the same haplotype (H1 which contains HM131183, HM131179, HM131180, AJ312041, AJ312042, AY795573, AF356845) appearing in France, Switzerland and the Czech Republic. All other haplotypes appear to be country specific, H2 contains AY795572; H3 contains Ham Ra7 and Ham Ra8; H4 contains Ham Ra 6; H5 contains HM131181, HM131177, HM131184, HM131182, HM131178, HM131176; H6 contains FJ469812; H7 contains AY713969; H8 contains AY713964; H9 contains AJ312044; H10 contains AJ312045; H11 contains AY713966; H12 contains FJ469809; H13 contains FJ469808; H14 contains AJ312046; H15 contains FJ469810; H16 contains FJ469811; H17 contains AY713973; H18 contains FJ469805 and FJ469806; H19 contains FJ469814 and FJ469821; H20 contains AJ312043; H21 contains FJ469819 and FJ469820.

Phylogenetic reconstruction showed the UK-specific sequences to have close affinity with populations of *T. franki* from France and clustered within a France-specific clade, but no other country specific clades were identified (Figure 4). Two unique haplotypes were identified from the three specimens with Ham Ra6 having its own individual haplotype and another being shared by Ham Ra7 and Ham Ra8 (GenBank: KJ775868-69). The molecular diversity of *T. franki* populations from each country appeared high with several different haplotypes being found within relatively small sample sizes (Table 3). Diversity within sequences from Switzerland showed considerable variation (S = 77; π = 0.02610) and five out of the six sequences analysed were unique haplotypes. This contrasted with sequences from France where only three haplotypes were found in the ten sequences sampled (S = 2; π = 0.00079). The high number of haplotypes within and between populations of *T. franki* was also illustrated in the haplotype network (Figure 5). Four distinct clades were revealed in the haplotype network, but no geographical specific lineages emerged as each clade contained a number of related haplotypes from different countries. Clade 1 contained the only haplotype shared between countries with haplotype 1 (H1) being shared by individuals from France (HM131183, HM131179, HM131180), Switzerland (AJ312041, AJ312042) and the Czech Republic (AY795573, AF356845). The UK-specific sequences were also found in clade 1 with haplotype 3 (H3) representing Ham Ra7 and 8 and haplotype 4 (H4) representing Ham Ra6. These haplotypes were unique and were not shared with other populations. However, the UK-specific sequences were most closely related to French sequences in haplotype 5 (H5) that contained HM131181, HM131177, HM131184, HM131182, HM131178, HM131176 and haplotype 2 (H2) that contained AY795572 (Figure 5). Although these data illustrate high population variation within and between *T. franki* populations, the sample sizes for each country are small. Further work with larger sample sizes would be required to provide in-depth insights into population differentiation and gene flow.

**Table 3:** **Molecular diversity of ITS1 haplotypes of *Trichobilharzia franki *** **from several European countries**

**Population**	**Number of sequences**	**S**	**H**	**Hd**	**K**	**π**
All	35	103	21	0.924 (SD ± 0.03)	33.2546	0.02013
UK (Hampshire)	3	1	2	1 (SD ± 0.5)	1	0.00118
France	10	2	3	0.6 (SD ± 0.131)	0.67	0.00079
Switzerland	6	77	5	0.93 (SD ± 0.122)	39.2	0.02610
Czech Republic	4	56	3	0.83 (SD ± 0.222)	29	0.01574
Poland	3	58	3	1 (SD ± 0.272)	39.67	0.01891
Iceland	10	81	7	0.93 (SD ± 0.062)	24.07	0.01791

Where S is number of polymorphic sites including indels; H is number of haplotypes; Hd is haplotype diversity; K is average number of differences between sequences and π is nucleotide diversity.

## Discussion

Utilising molecular techniques described by Jouet *et al*. [24], our study has provided the first detailed record of a *Trichobilharzia* parasite in the UK. The morphological description of *T. franki* supported by scanning electron microscopy observations, shows that the cercariae have considerable similarity to other *Trichobilharzia* species, particularly *T. regenti*. A molecular approach was therefore considered essential for identification. Phylogenetic analysis of the LSU and analysis of *p*–distance demonstrated that the UK-specific sequences corresponded to *T. franki,* showing the power of this ribosomal marker for species-specific identification and high levels of phylogenetic resolution as in other studies of avian schistosomatids [1,24]. However, there is clear evidence of a close relationship between *T. franki* and *T. querquedulae,* as also highlighted by Brant and Loker [1] and Jouet *et al*. [24]. *Trichobilharzia querquedulae* could be either a very close sister taxon, part of a species complex in which a number of *Trichobilharzia* species are very closely related and form the “*T. franki*” complex [24], or simply be a genetically distinct population of *T. franki* and not a species in its own right [24]. Such confounding factors on the identity of a species could be problematic for phylogenetic analysis and subsequent epidemiological surveys. Although the UK parasite was identified as *T. franki*, other molecular markers could be considered for identification of other species and genera of non-*Schistosoma* schistosomatids, to provide a comprehensive molecular phylogenetic framework for avian schistosomatid parasites [24-26].

Both the phylogenetic and haplotype network analysis of the ITS1 fragment from the UK samples show a close genetic relationship with French populations (Figures 4 and 5). The presence of *Trichobilharzia* spp. and occurrence of CD have been known in France for several decades [2,24] and, due to close proximity of France to the UK and the movement of migratory waterfowl between the two countries, it could be argued that although some differentiation between populations of *T. franki* has occurred, some gene flow is likely to take place. The occurrence of gene flow between countries was also illustrated in the haplotype network analysis due to H1 being shared between France, Switzerland and the Czech Republic (Figure 5). This is a clear indication that the distribution and prevalence of CD is intimately linked with the movement of waterfowl, and *T. franki* populations are moving around Europe, probably as a result of the migratory patterns of their definitive Anseriformes hosts (ducks, geese and swans) [1,9,24]. Brant and Loker [1] discussed the occurrence of genetically related individuals of *T. physellae* and *T. querquedulae* collected from snails along the major avian migratory flyways in the USA ranging from latitudes as distant as Alaska to Louisiana and Florida. This is probably also true for *T. franki* in the UK and the rest of Europe as some birds, such as swans and geese, migrate from Iceland to overwinter in the UK and mainland Europe from October to April, which would result in the movement of parasites within and between countries, reducing the level of genetic differentiation between *T. franki* populations [9]. It can be concluded from the current study that the collected parasites are closely related to populations in France, and it is highly likely that *T. franki* in Hampshire was brought to the UK by waterfowl migrating from France. Although the specimens used in this study were collected from a single site, it is likely that there are several different genetically unique populations of *T. franki* throughout the UK, particularly given the wide range of migratory waterfowl that visit the UK.

Little is known about the distribution of *T. franki* and other *Trichobilharzia* species in the UK and further investigation is required to identify the association of migratory birds and parasite distribution in order to fully understand the health risk of this parasite to the UK human population. Cercarial dermatitis is considered a global risk to human health and several authors have argued that the disease in Europe should be considered as re-emerging rather than a new threat [2,3,27]. This is partly because of changes in human activity, especially recreational use of water bodies over the previous two decades. There has been a long history and understanding of CD in mainland Europe and cases are frequently reported (reviewed by Soldánová *et al*. [27]). In the UK, however, the risk of infection and epidemiology of CD are relatively unknown and the disease has to be considered a potential re-emerging disease. This is mainly due to a distinct lack of knowledge of the occurrence of agents of CD and their abundance, but also to the lack of recorded cases with only a few cases being reported to physicians because symptoms can be pathologically benign and confused with other allergic reactions or insect bites [5,28]. In addition to human factors, it is important to note that recent climatic changes could also influence the epidemiology of CD [29]. All fluke infections in snails are climate/temperature sensitive with higher ambient temperatures tending to, not only result in higher cercarial emission, but also lead to higher snail numbers increasing the potential number of intermediate hosts and thus the risk of infection [29]. Climate change can also affect the distribution of definitive hosts, altering migratory routes and thus enabling infected bird species to visit the UK, which may not have done so before [29,30]. Although the UK has many native species of snail intermediate hosts of avian schistosomatids, it is possible that populations of introduced snail vectors could establish themselves in countries such as the UK that were previously too cold to sustain them; this has been observed with snail hosts of other schistosomatids. For example, *Biomphalaria tenagophila*, a snail vector of *Schistosoma mansoni,* which is a major agent of hepato-intestinal human schistosomiasis in Africa, has recently become established in Romania [31].

## Conclusion

This current study describes the first specific and detailed record of *T. franki* in the UK, predominately based on molecular evidence, and illustrates the close relationship between the population sampled in this study and populations found throughout France. The relationship between the parasite populations is likely to be a result of the movement of parasites between countries due to migratory routes of waterfowl. Therefore, it is crucial to understand the role of waterfowl in the epidemiology of CD, and to apply appropriate tools to identify *Trichobilharzia* species in order to assess the risk of CD outbreaks and their impact on human health in the UK. This is particularly pertinent in view of the distinct lack of knowledge of the diversity of zoonotic flukes and their snail intermediate hosts within the UK and mainland Europe, especially when considering the potential impact to public health [32,33].

## Abbreviations

CD: Cercarial dermatitis; G: Gamma distribution; H: Haplotype; Hd: Haplotype diversity; HKY: Hasegawa-Kishino-Yano substitution model; ITS1: Internal transcribed region 1; K2: Kimura 2-parameter model; LSU: Large subunit of ribosomal gene; ML: Maximum likelihood; MP: Maximum parsimony; NJ: Neighbour joining; PCR: Polymerase chain reaction; π: Nucleotide diversity.

## Competing interests

The authors declare that they have no competing interests.

## Authors’ contribution

SPL, JD, RC, AJW, RSK were involved in conceiving the project and wrote the manuscript. SPL, RSK, JD and RC performed the field collection and RC identified snails. RSK morphologically identified and described the furcocercariae. SPL, RML and JD performed the molecular laboratory work and phylogenetic analysis. SPL and RML performed the bioinformatics and population level analysis. All authors read and approved the final version of the manuscript.
